# Intra-arterial infusion chemotherapy versus isolated upper abdominal perfusion for advanced pancreatic cancer: a retrospective cohort study on 454 patients

**DOI:** 10.1007/s00432-019-03019-6

**Published:** 2019-09-10

**Authors:** Karl R. Aigner, Sabine Gailhofer, Emir Selak, Kornelia Aigner

**Affiliations:** Department of Surgical Oncology, Krankenhausstr. 3a, 84489 Burghausen, Germany

**Keywords:** Pancreatic cancer, Regional chemotherapy, Isolated perfusion, Intra-arterial infusion, Quality of life

## Abstract

**Purpose:**

The treatment of pancreatic carcinoma remains a challenge as prognosis is poor, even if confined to a single anatomical region. A regional treatment of pancreatic cancer with high drug concentrations at the tumor site may increase response behaviour. Intra-arterial administration of drugs generates homogenous drug distribution throughout the entire tumor volume.

**Methods:**

We report on treatment outcome of 454 patients with advanced pancreatic carcinoma (WHO stage III: 174 patients, WHO stage IV: 280 patients). Patients have been separated to two different treatment protocols. The first group (*n* = 233 patients) has been treated via angiographically placed celiac axis catheters. The second group (*n* = 221 patients) had upper abdominal perfusion (UAP) with stopflow balloon catheters in aorta and vena cava. Both groups have been treated with a combination of cisplatin, adriamycin and mitomycin.

**Results:**

For stage III pancreatic cancer, median survival rates of 8 and 12 months were reached with IA and UAP treatment, respectively. For stage IV pancreatic cancer, median survival rates of 7 and 8.5 months were reached with IA and UAP treatment, respectively. Resolution of ascites has been reached in all cases by UAP treatment. Toxicity was generally mild, WHO grade I or II, toxicity grade III or IV was only noted in patients with severe systemic pretreatment. The techniques, survival data and detailed results are demonstrated.

**Conclusions:**

Responsiveness of pancreatic cancer to regional chemotherapy is drug exposure dependent. The isolated perfusion procedure is superior to intra-arterial infusion in survival times.

**Electronic supplementary material:**

The online version of this article (10.1007/s00432-019-03019-6) contains supplementary material, which is available to authorized users.

## Introduction

The therapy of pancreatic cancer remains a major challenge in cancer treatment, which is clearly reflected in consistent poor survival rates. As mortality rates match the growing incidence of pancreatic cancer, 5-year survival rates have not exceeded 5%, which is alarming and requires intensive research and new methods and techniques, especially for unresectable cancers (Siegel et al. 2018). Gemcitabine as a single agent has been the front-line chemotherapy for pancreatic cancer since the 1990s, showing a survival advantage of 2 months. Various combination therapies with gemcitabine have been disappointing, except the tyrosine kinase inhibitor erlotinib plus gemcitabine, which elevated the 1-year survival rate from 17 to 23% (Moore et al. 2007; National Comprehensive Cancer Network 2018; Ducreux et al. 2015; El Rassy et al. 2017). At present, combination therapies with FOLFIRINOX, nab-paclitaxel, gemcitabine or nanoliposomal irinotecan in combination with 5-FU and leucovorin are the preferred first-line regimens (Ghosn et al. 2016; Wang-Gillam et al. 2016; Oettle et al. 2014; Gill et al. 2016; Zaniboni et al. 2012; Assaf et al. 2011; Kim 2016; Ko et al. 2013; Glassman et al. 2018). Recently, tumor infiltrating macrophages (TAMs) have been identified to be a main hindrance for a lasting effect of gemcitabine and are the main reason for immunosuppressive conditions (Halbrook et al. 2019; Wu et al. 2019). There is limited success in the use of immunotherapy in the treatment of pancreatic cancer, which might be due to an unfavorable tumor microenvironment (Torphy et al. 2018). In numerous recent studies investigating new therapies or therapy combinations for pancreatic cancer, the erstwhile primary treatment objective of overall survival has been replaced by surrogate endpoints such as tumor response or progression-free survival (PFS), which, however, turned out to have no meaningful impact on overall survival. In contrast, additional side effects from systemic chemotherapy are accompanied by high financial costs as well as disrupted quality of life during and after treatment, without any survival advantage. Thus, clinical efficacy in relation to toxicity should be considered in the decision-making process. Additionally, ascites is a negative prognostic marker, and, to date, no adequate treatment is available (Baretti et al. 2019). We herein present a technique that directly targets the cancer in the affected body segment while keeping the rest of the body unaffected and therefore avoiding undesirable adverse events.

## Methods

### Patient description

This is a retrospective observational cohort study. We reviewed the medical records of 454 patients with unresectable pancreatic cancer who were treated between 1987 and 2017. The median age of all patients was 61 years (range 18–80). There were 192 female and 262 male patients; 391 patients had adenocarcinoma; 7 patients had endocrine carcinoma and 56 patients had an unknown histological status. Regarding classification, 174 patients were classified stage III, and 280 patients were classified stage IV. Furthermore, 265 patients had no prior treatment, and 189 patients had prior treatment with systemic chemotherapy, chemoembolization, and/or irradiation. Observation time was at least 17 months (Table 1).

**Table 1:** Pancreatic cancer patient demographics, 1987–2017

	Total (*N* = 454)	III (*n* = 174)				IV (*n* = 280)			
		i.a. chemo	UAP	Chi-squared test	*p* value	i.a. chemo	UAP	Chi-squared test	*p* value
*n*	454	95	79	–	–	138	142	–	–
Male	267	43	48	48.44	< 0.01	79	92	104.68	< 0.01
Female	187	52	31	40.60	< 0.01	59	50	42.96	< 0.01
Tumor size > 5 cm	207	2	9	5.87	–	44	152	157.88	< 0.01
Tumor size > 8 cm	89	1	8	6.64	–	17	63	38.37	< 0.01
Adeno ca	391	91	63	136.66	< 0.01	114	123	200.64	< 0.01
Endocrine ca	7	0	0	–	–	3	4	0.29	–
n.a.	56	9	11	2.89	< 0.1	17	19	4.68	< 0.05
No metastases	175	95	79	–	–	0	0	–	–
1 organ metastasized	123	0	0	–	–	83	47	65.53	< 0.01
2 organs metastasized	111	0	0	–	–	46	53	35.18	< 0.01
3 organs metastasized	33	0	0	–	–	8	29	13.78	< 0.01
4+ organs metastasized	15	0	0	–	–	1	13	9.75	< 0.01
Peritoneal carcinosis	79	0	1	–	–	16	52	28.35	< 0.01
Ascites (medium to severe)	36	5	6	1.02	< 0.9	9	16	3.72	< 0.1
Pretreated with systemic chemotherapy	174	8	24	13.89	< 0.01	42	55	34.37	< 0.01
Gemcitabine	51	2	5	2.04	–	17	27	8.41	< 0.01
5-FU (+ leucovorin)	29	3	9	4.52	–	8	9	1.06	< 0.9
FOLFIRINOX	8	1	0	0.83	–	0	7	6.66	–
FOLFOX	2	0	0	–	–	0	2	1.93	–
Erlotinib	8	0	1	1.19	–	0	7	6.66	–
Cisplatin	6	0	0		–	2	4	0.71	–
Others (irinotecan, PEFG, POO, navelbine, ardalan, novantron, GEMOX, Nab-paclitaxel, COSS96, fluroblastin, farnesylproteintransferase, FAM, endoxan, ELF, ACO, Etoposid, paclitaxel, IFNalpha, lok.Hyperthermia)	85	6	13	5.35	< 0.025	32	34	15.57	< 0.01
Regional chemotherapy	454	95	79	–	–	138	142	–	–
Cisplatin/adriamycin/mitomycin	454	95	79	–	–	138	142	–	–
Additional cycles with gemcitabine	60	0	27	–	–	0	33	–	–

Patient groups similarity was testet according to Pearson’s Chi-square test. Patient characteristics with *p* values < 0.05 were accepted as equably distributed. Characteristics that were found in less than five patients per group could not be tested

Investigations were performed in compliance with the principles of good clinical practice outlined in the Declaration of Helsinki and federal guidelines and were approved by the Medias Institutional Review Committee. Informed consent was obtained from each participant or participant’s guardian.

The mode of drug administration was either intra-arterial infusion through an angiographic catheter alone (233 patients) or a combination of intra-arterial infusion and at least one upper abdominal hypoxic perfusion with chemofiltration (UAP-F; 221 patients).

### Technique of upper abdominal hypoxic perfusion with chemofiltration (UAP-F)

**Fig. 1:**
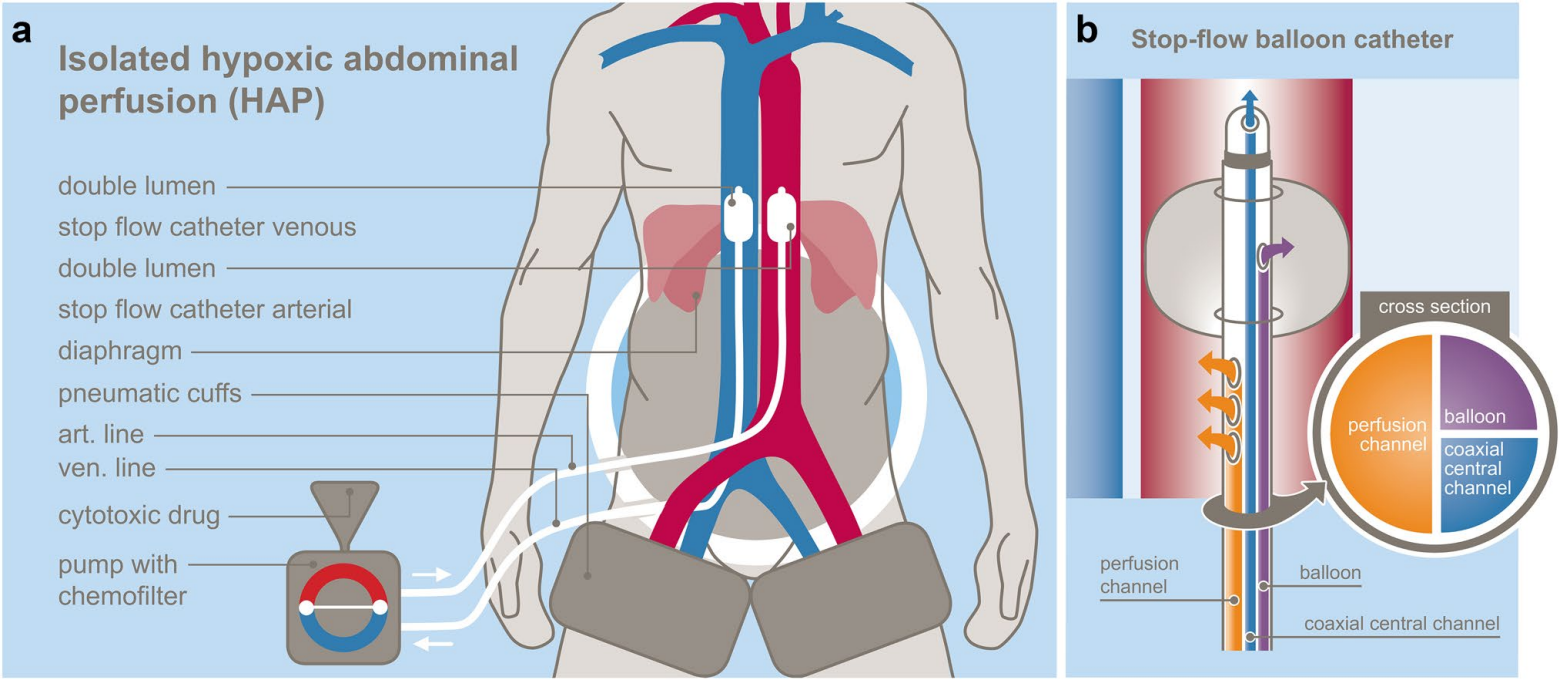
**a** Scheme of UAP-F technique. **b** Stop-flow balloon catheter

Upper abdominal perfusion is a method performed in two steps, where the first step is the stop-flow procedure and the second step is the isolated hypoxic abdominal perfusion (Fig. 1a). Both steps are performed with stop-flow balloon catheters that are first inserted through the femoral artery and vein in the groin area and threaded into the vena cava and aorta. The venous balloon is placed beneath the diaphragm. Its position is controlled through radiography by temporarily inflating it with contrast medium, followed by deflation until the therapy is started.

Stop-flow balloon catheters consist of three channels: one for the isolated perfusion, one for inflation of the balloon, and one coaxial channel exiting at the tip above the balloon for potential insertion of a guidewire and/or injection of chemotherapeutics as well as monitoring of arterial blood pressure during the procedure (Fig. 1b).

For the first step (stop flow), the balloon in the aorta is positioned right beneath the celiac trunk and inflated, and its correct position is controlled by radiography with intra-arterial contrast medium. Chemotherapy is infused for 1 min under high oxygenation while an outflow-block of the liver veins is contemporarily established by inflating the venous balloon. Because of the infra-celiac aortic balloon block, the first-pass flow of chemotherapeutics exits exclusively through the celiac trunk that supplies the tumor region. Thereafter, the aortic balloon is immediately slipped upstream in the aorta and placed right beneath the diaphragm. At this point, the hypoxic part of the procedure begins. Thus, both balloons stop the blood flow, and a high drug concentration is maintained in the distribution area of the celiac trunk in the upper abdominal region for 5 min.

During the second step, the isolated hypoxic abdominal perfusion is started via the side holes in the perfusion channel of the catheter beneath each balloon. After 5 min of perfusion with the high drug concentrations in the whole abdominal region, another 5 min of perfusion with additional chemofiltration is maintained in order to lower the cytostatic load before deflating the balloons and continuing chemofiltration until a substitution volume of 4 L is reached (Fig. 1a).

The isolated hypoxic abdominal perfusion (HAP) step has been applied 520 times for 221 patients, upon which 318 procedures comprised the stop-flow interval and HAP, and 202 procedures were application of HAP only.

### Intra-arterial infusion chemotherapy via angiographic catheters in the celiac trunk

Intra-arterial infusion chemotherapy is performed in local anesthesia via a Sidewinder angiocatheter. It has been administered 977 times for 233 patients; in 26 instances, additional chemofiltration was applied, and in 231 procedures, it was combined with chemoembolization with starch microspheres.

### Treatment cycles and drug regimen

Drug combinations for UAP and HAP were 50 mg cisplatin, 30 mg adriamycin and 15 mg mitomycin C for an average 70 kg patient. Adriamycin and mitomycin C have been shown to have a particular strong cytotoxic effect under hypoxic conditions while cisplatin works equally well under aerobic and hypoxic conditions (Teicher et al. 1981). For i.a. chemotherapy infusion via an angiocatheter, a drug combination of 30 mg cisplatin, 2 × 15 mg adriamycin and 10 mg mitomycin C was administered for 5 min each on four consecutive days. In addition, 29 patients also received cycles with 600–1000 mg gemcitabine and cisplatin. Depending on disease extension and response, 2–10 treatment cycles in 3 weeks intervals each were administered. The median number of cycles was E4. One exceptional patient received 26 cycles over a 6-year period.

### Blood sampling methods for cisplatin plasma concentration measurements

A series of cisplatin plasma concentration measurements has been investigated. The measurements were obtained during and after intra-arterial infusion of CDDP (cisplatin) through the coaxial channel of the balloon catheter. For intra-arterial infusion, the duration was chosen according to the infusion duration in tumors of the head and neck, which was 5–7 min (Aigner et al. 2018, 2019). Its concentrations were measured at minutes 1, 2 and 3, respectively, with measurements collected at 2-min intervals. The arterial blood samples were taken with a Sidewinder-II catheter from the common hepatic artery supplying the tumor. The venous blood samples were taken from a central venous catheter. The blood samples were centrifuged and plasma drug concentrations were measured.

### Criteria for response and adverse events

Tumor responses were assessed in accordance with Response Evaluation Criteria in Solid Tumors (RECIST version 1.1) at 2 weeks after every second treatment cycle. Responses were evaluated by computed tomography (CT), magnetic resonance imaging (MRI), and positron emission tomography (PET). Pain controlled by < 50% analgesic administration at 20 days after treatment was considered objective pain relief. Adverse events were assessed according to the common terminology criteria for adverse events of the National Cancer Institute.

### Statistical analysis

Statistics were calculated with 95% confidence limits. Survival times were estimated using the Kaplan–Meier product limit estimator, and follow-up for surviving patients was minimum 10 months, with a median follow-up of 39 months. Survival times were stratified according to clinical variables that may affect survival, and log-rank tests were used to verify significance. Statistical analyses were performed by using MediasStat software version 28.5.14.

## Results

### Drug concentrations

**Fig. 2:**
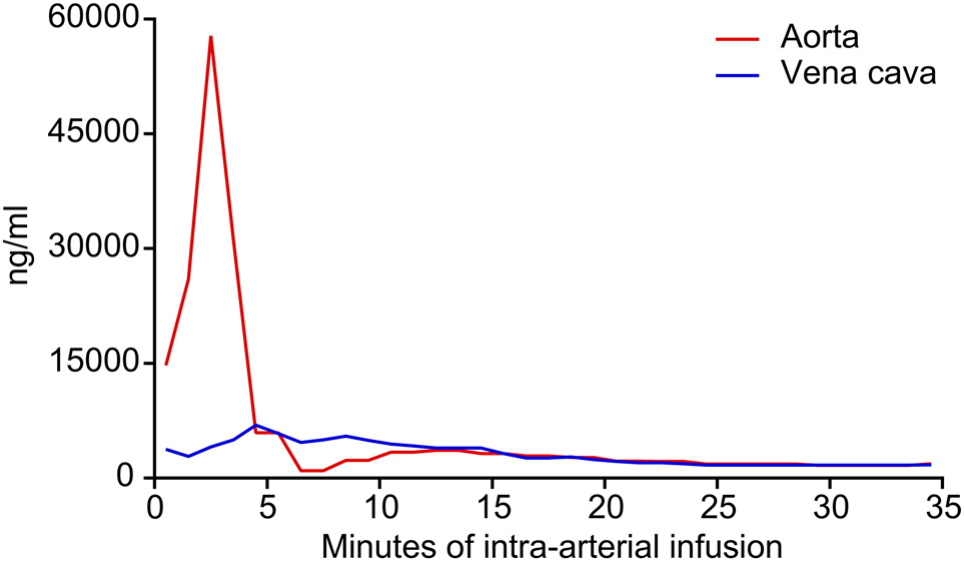
Cisplatin concentrations during intra-arterial infusion

During the short-term intra-arterial infusion of chemotherapy through the coaxial catheter channel, cisplatin concentrations in the tumor supplying artery were shown to reach levels of up to 60,000 ng/mL. Venous cisplatin levels remained low both during and after the infusion (Fig. 2).

### Response rates

Resolution of ascites was achieved in 33 out of 36 cases of medium to severe ascites with the upper abdominal perfusion with chemofiltration technique (UAP-F). Response rates for tumor markers were as follows: 8.7% complete response, 53.6% partial response, 13% stable disease and 24.6% progressive disease. Responses each lasted at least 8 weeks.

### Overall survival

**Fig. 3:**
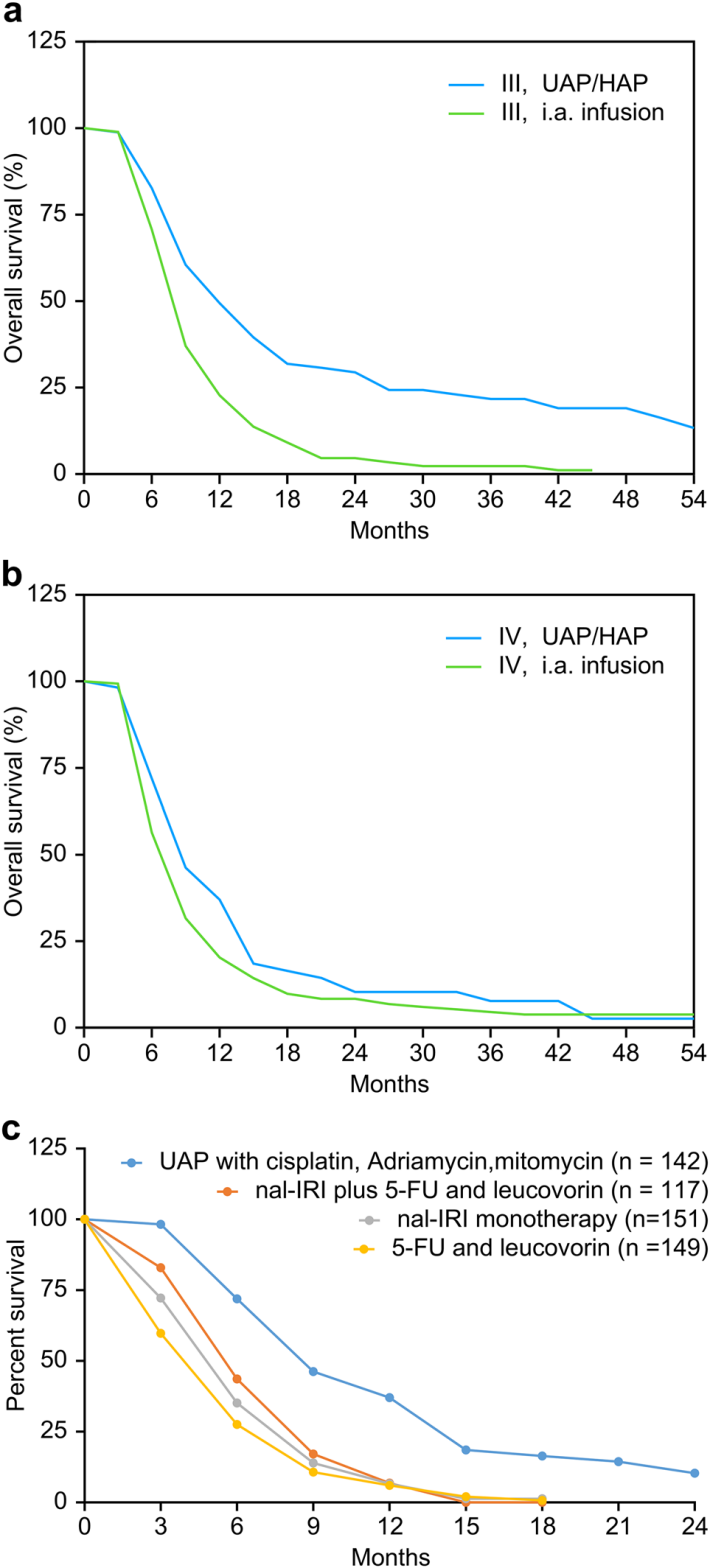
**a** Kaplan–Meier estimate of survival comparing intra-arterial infusion with UAP-F in stage III pancreatic cancer. **b** Kaplan–Meier estimate of survival comparing intra-arterial infusion versus UAP-F in stage IV pancreatic cancer. **c** Metastatic carcinoma of the pancreas treated with upper abdominal hypoxic perfusion (UAP-F)

Overall survival was estimated using the Kaplan–Meier calculations for patients with stage III and stage IV disease. Patients were grouped according to the administered regional chemotherapy treatment technique, which either was intra-arterial infusion via angiographic catheters (i.a. infusion) or a combination of isolated upper abdominal stop-flow perfusion and hypoxic abdominal perfusion with chemofiltration (UAP-F). In stage III cancers, median survival times of 7.6 and 12.1 months were reached in patients treated with i. a. infusion and UAP-F, respectively. In stage IV cancers, median survival times of 6.6 and 8.7 months were reached in patients treated with i. a. infusion and UAP-F, respectively (Fig. 3a). All results were statistically significant with *p* < 0.05 and *p* < 0.01 for stage III and stage IV, respectively. Clinical relevance is particularly shown in the benefit of UAP-F at stage III. 1-year survival was 22.8% and 49.4% for stage III patients treated with intra-arterial infusion and UAP-F, respectively. 3-year survival was 2.3% and 21.7% for stage III patients treated with i. a. infusion and UAP-F, respectively. 1-year survival was 20.3% and 37.0% for stage IV patients treated with i. a. infusion and UAP-F, respectively. 3-year survival was 4.5% and 7.7% for stage IV patients treated with i. a. infusion and UAP-F.

### Adverse events from UAP-F

Side-effects were generally mild. Bone marrow suppression was WHO grade 1–2 in treatment-naïve patients. Neurotoxicity from regional therapy was never observed (Table 2).

**Table 2:** Adverse events

	*n*	(%)	i. a. chemotherapy	%	UAP	%
	454	100	233	100	221	100
Bone marrow suppression grades 1–2	77	17	20	8.6	57	25.8
Bone marrow suppression grades 3–4 (only patients with multiple lines of previous systemic chemotherapy)	23	5	4	1.7	19	8.6
Postoperative wound infection	5	1	0	0	5	2.3
Postoperative thrombosis	5	1	0	0	5	2.3
Temporary lymph fistula in the groin	136	30	0	0	136	61.5
Renal toxicity	50	11	0	0	50	22.6
Nausea grade 1	159	35	70	30	89	40.2
Nausea grade 2	68	15	33	14	35	15.8
Vomiting grade 2	45	10	21	9	24	10.9
Diarrhea	23	5	2	0.8	21	9.5
Neurotoxicity (only patients with multiple lines of previous systemic chemotherapy)	14	3	7	3	7	3.1
Incomplete hair loss	59	13	31	13.3	28	12.7
Fatigue	47	10.4	21	9	26	11.8

## Discussion

The dose-dependent toxicity of cytostatic drugs on tumors is a familiar and well-established principle. In the clinical application of systemic chemotherapy, however, the required cytostatic exposure of solid tumors is limited by escalating systemic toxicity. Everything achieved so far in terms of survival and quality of life in the treatment of pancreatic cancer is at best paltry yet extremely costly and often associated with a loss of quality of life. It has been established time and again that patients already suffering from this highly malignant disease should not be expected to bear the burdens of therapy-related side effects on top of everything else (El-Rayes et al. 2003).

The poor responsiveness of pancreatic primary tumors as compared to their well responsive liver metastases is due to the difference in vascularization. During surgery, pancreatic carcinomas appear nearly avascular on their cut surface, i.e., without vascularization, whereas liver metastases from the same tumor have an excellent blood supply, as seen when methylene or indigo carmine blue is injected through the hepatic artery for staining (Aigner et al. 2016). A better response of liver metastases was reported in some studies on intra-arterial chemotherapy (Beger et al. 1999; Aigner and Gailhofer 2005; Ishikawa et al. 1997).

Studies on intra-arterial chemotherapy have so far been quite heterogeneous and include the use of different chemotherapeutic agents at completely different doses and application times. However, despite the wide range of applications in all the studies, survival times are generally better, and toxicity is lower than described for systemic chemotherapy. There are two randomized phase III trials comparing systemic chemotherapy with regional chemotherapy in advanced pancreatic cancer (Aigner et al. 1998; Cantore et al. 2003) and one meta-analysis on six randomized studies (Liu et al. 2012). In all studies, regardless of the different drug combinations, significantly better response and overall survival have been noted in the intra-arterial infusion arm, whereas toxicity was significantly increased in the systemic arm.

In the present retrospective comparative cohort study of two treatment modalities, all 454 patients were treated by the same team. During the first two decades, the treatment of choice was intra-arterial infusion, while during the last decade, upper abdominal and abdominal perfusion with chemofiltration were incorporated into the therapeutic protocol.

A limitation of this study is the lack of randomization. However, patients in both arms are comparable in terms of age, histology and primary tumor size but differ in the number of metastasized locations, with the UAP group showing more affected locations. All therapies were performed by the same group. There is a big difference in the clinical outcome in both stages III and IV when comparing intra-arterial infusion with upper abdominal perfusion and chemofiltration, indicating a superiority of higher cytostatic exposure in isolated perfusion. In stage III disease, there is a big difference in median survival of 7.6 months after i. a. infusion versus 12.1 months after UAP-F (*p* value < 0.05). In addition, in stage IV disease, which had the majority of bulky tumors, there is a difference between i. a. infusion versus UAP-F, with survival times of 6.6 and 8.7 months, respectively, with a *p* value of 0.01. Most interestingly, 3-year survival in the UAP-F group was 21.7%, while the intra-arterially treated stage III patients only achieved 2.3% survival after 3 years. Even in stage IV disease, there was still a 7.7% 3-year survival in the UAP-F group. These results underline the steep dose- and concentration–response behavior in chemotherapy (Frei and Canellos 1980).

The overall survival of 57 stage IV patients treated with upper abdominal perfusion and chemofiltration reveals superior survival at 6, 9, 12 and 18 months compared with nal-IRI plus 5-FU and leucovorin, nal-IRI monotherapy and 5-FU and leucovorin therapy from the Napoli-1 study (Fig. 3c) (Wang-Gillam et al. 2016). Regardless of a lack of randomization in the comparison of a selection of highly advanced cases to be treated, differences between the two treatment modalities—systemic chemotherapy and isolation perfusion—are evident. On the other hand, a major aspect is the extremely improved quality of life following regional chemotherapy and a lack of stage 3 and 4 toxicities. Most patients report no subjective side-effects at all.

Pancreatic cancer therapies with upper abdominal perfusion and chemofiltration have the definite advantage of a high first-pass effect from the selective infusion of highly concentrated chemotherapeutics through the celiac trunk followed by the stop-flow procedure, which is comparable with transarterial chemoembolisation (TACE), and the subsequent isolated perfusion, all together resulting in much higher local drug exposure.

HAP without chemofiltration has been described by other groups in the literature with case numbers of about 20 (17–22) patients with consistently poor results and high toxicity (Lorenz et al. 1998; Van Ijken et al. 2004; Meyer et al. 2006). The reason for the weak effect and the bad results are probably different application methods, especially the administration of drugs in the hypoxic perfusion circuit, and not in the hyperoxygenated environment before occlusion of the aortic balloon. Another major reason for better response is, that in upper abdominal perfusion the drugs are injected into a much smaller volume of blood and thus the effective exposure is higher. A further study on HAP but with chemofiltration concludes that the method is effective and the complication rate low (Guadagni et al. 2007). Regional chemotherapy offers great therapeutic potential, much of which is still untapped, while being less detrimental on the patients’ quality of life. While progress is definitely being made, it is still a painstaking, protracted and laborious process. The response rates even for very advanced cases, the overall survival and the adverse events profile show that UAP is a feasible method with promising results and deserves a multicenter controlled phase-III-study to be further evaluated.

## Conclusions

Responsiveness of pancreatic cancer to regional chemotherapy is drug exposure dependent. The isolated perfusion procedure is superior to intra-arterial infusion in survival times.

## Electronic supplementary material

Below is the link to the electronic supplementary material.

Supplementary material 1 (DOCX 12 kb)
