# Supplementary material for: Intra-arterial infusion chemotherapy versus isolated upper abdominal perfusion for advanced pancreatic cancer: a retrospective cohort study on 454 patients
Source: J Cancer Res Clin Oncol. 2019 Sep 10;145(11):2855–62. doi: 10.1007/s00432-019-03019-6 (PMC6800855; doi:10.1007/s00432-019-03019-6)
Supplement: Supplementary file 1 — Supplementary material 1 (DOCX 12 kb) [file 432_2019_3019_MOESM1_ESM.docx]

Supplementary file

Survival times of various treatment modalities

| Months | III, UAP/HAP | III, i.a. infusion | IV, UAP/HAP | IV, i.a. infusion |
| --- | --- | --- | --- | --- |
| 6 | 82.7 | 70.7 | 71.9 | 56.4 |
| 12 | 49.4 | 22.8 | 37 | 20.3 |
| 18 | 31.9 | 9.1 | 16.4 | 9.8 |
| 24 | 29.4 | 4.6 | 10.3 | 8.3 |
| 36 | 21.7 | 2.3 | 7.7 | 4.5 |
